# Prioritising Profits Over Public Health? Why UK Commercial Milk Formula Marketing Regulations Need to be Strengthened and Enforced, Not Weakened

**DOI:** 10.1111/mcn.70002

**Published:** 2025-02-13

**Authors:** Victoria Sibson, Marko Kerac, Robert Boyle, Amandine Garde, Andrea Gideon, Shereen Fisher, Catherine Pereira‐Kotze

**Affiliations:** ^1^ First Steps Nutrition Trust London UK; ^2^ London School of Hygiene and Tropical Medicine London UK; ^3^ National Heart and Lung Institute, Imperial College London London UK; ^4^ UK Law and Non‐Communicable Diseases Research Unit, School of Law and Social Justice University of Liverpool Liverpool UK; ^5^ UK Committee for UNICEF (UNICEF UK) Baby Friendly Initiative London UK

## 1 Infant and Young Child Feeding in the UK

Current UK public health recommendations for feeding in the first years of life align with those of the World Health Organization and recognise breastfeeding as optimal for infant and maternal health. Exclusive breastfeeding for the first 6 months is therefore recommended, and so is continued breastfeeding, alongside a diet of appropriate, nutritious complementary foods, to 2 years and beyond (SACN 2018; World Health Organization [WHO] 2023a). Beyond health benefits, breastfeeding also has environmental and climate benefits (Joffe et al. 2019).

Most UK women want to breastfeed (McAndrew et al. 2012), and most start breastfeeding (NHS Digital 2024). However, too few receive the support they need to meet their breastfeeding goals (McAndrew et al. 2012). One major factor undermining their efforts is aggressive and misleading marketing of breastmilk substitutes (including commercial milk formulas, bottles and teats) (World Health Organization [WHO] and the United Nations Children's Fund (UNICEF) 2022; Rollins et al. 2023). Such marketing contributes to the widespread use of commercial milk formulas in the first weeks and months of life for most UK infants (NHS Digital 2024). This is unfortunate: whilst infant formula is safe and suitable to support adequate growth and development, it cannot match full health benefits of breastfeeding (NHS 2023). Other commercial milk formulas are unnecessary for healthy children (NHS 2023; Sibson and Westland 2024; Childs et al. 2024). Effective controls on the marketing of all commercial milk formulas are thus necessary to ensure women and families can make properly informed, independent decisions on how they feed their babies and young children, protected from undue commercial influence.

Marketing controls are outlined in the global policy framework: the International Code of the Marketing of Breastmilk Substitutes and its subsequent World Health Assembly Resolutions (‘the Code’) (World Health Organization [WHO] 2022), supplemented by recent guidance on digital marketing (World Health Organization [WHO] 2023b). The Code makes clear that typical marketing practices cannot be applied to commercial milk formulas: “*… in view of the vulnerability of infants in the early months of life and the risks involved in inappropriate feeding practices, including the unnecessary and improper use of breast‐milk substitutes, the marketing of breast‐milk substitutes requires special treatment, which makes usual marketing practices unsuitable for these products*” (World Health Organization [WHO] 2022). The Code not only protects breastfeeding, but it also ensures safe and appropriate formula feeding where commercial milk formula is used (UNICEF 2024) (see box).

**Box: How ‘the Code’ protects all infants and young children, regardless of how they are fed** (page 9, from “Countering Industry Arguments against Code Implementation: Evidence and Rights‐Based Responses”, UNICEF 2024).

- “It recognizes that when mothers do not breastfeed, there is a legitimate market for formula and baby feeding products and that these products should be made accessible to those who need them.
- While the Code ensures that breastmilk substitutes are not marketed or distributed in ways that may discourage breastfeeding, it also ensures that decisions on the use of breastmilk substitutes should be based on accurate information and counselling.
- The Code further ensures that product labels carry necessary warnings and directions for safe preparation.”

## 2 Weak and Disregarded UK Regulations on Commercial Milk Formula Labelling Undermine Safe and Appropriate Infant and Young Child Feeding

Kamata and others have exposed the shockingly poor compliance of commercial milk formula companies with UK marketing regulations on the labelling of infant and follow‐on formula (Kamata et al. 2025). This is happening despite clear guidance from the Department of Health and Social Care (Department of Health and Social Care [DHSC] 2024) and even though these domestic regulations are already less stringent than the international minimum standards outlined in ‘the Code’ (World Health Organization [WHO] 2022). Formula companies exploit weaknesses in the UK's overburdened and under resourced enforcement system (Conway et al. 2023; Baby Feeding Law Group [BFLG‐UK] 2025) to maximise their sales, whilst undermining breastfeeding as well as safe and appropriate formula feeding (UNICEF 2024). Whilst companies benefit from the current regulatory environment, the taxpayer is ultimately burdened with the long‐term costs of poor health outcomes that arise from suboptimal infant and young feeding.

## 3 Companies Use Misleading Marketing to Push Up the Price of Infant Formula

Kamata's work was done well before the 19 February 2025 statutory deadline by which the UK Government watchdog, the Competition and Markets Authority (CMA), is due to publish the results of a ‘market study’ into the UK's infant and follow‐on formula market (CMA 2024a). Using its powers to request disclosure, the CMA has obtained market data from formula companies and retailers to accurately describe the current structure of the UK infant and follow‐on formula market and consider possibilities for “how to drive better market outcomes for parents and carers” (CMA 2024b).

The CMA ‘interim report’ published in November 2024 shows how the commercial milk formula market in the UK has become hyper concentrated, being dominated by a small number of companies. The CMA has reported limited retail competition, widespread non‐compliance with marketing regulations and a disproportionate reliance on brand‐building and inaccurate product differentiation by companies in attempts to capture new customers (CMA 2024b).

This CMA market study is also consistent with previous CMA work, which found that ‘big brand’ formula companies were profiteering during the ‘cost of living’ crisis (CMA 2023), even if this has meant that infant formula has become unaffordable for some families on low incomes (Haydon and Brindsen 2024). This exposé of company profiteering on infant formula was made possible because of price data the UK charity First Steps Nutrition Trust have been collecting, reporting and advocating on since 2018 (All‐Party Parliamentary Group on Infant Feeding and Inequalities 2018) and which continues to be reported every 6 months (First Steps Nutrition Trust 2024).

The CMA's final market study report will include recommendations to the Government on how to address unjustifiably high infant formula prices which threaten infant health.

## 4 Commercial Milk Formula Marketing Regulations Need to be Stronger and Enforced, Not Weaker, to Bring Down Prices and Protect the Health of All Infants and Young Children, However They Are Fed

The CMA investigation represents an unprecedented opportunity to fix the myriad problems with the UK's commercial milk formula market to the benefit of parents/carers and ultimately their babies and young children. But in doing so, it is imperative that public health concerns are placed at the centre of the process.

The CMA's interim report suggests some sound policy solutions, e.g. price controls, standardised product labelling and improved provision of DHSC‐approved information to parents. In contrast, the proposal to encourage competition between companies through price promotions is alarming. For non‐essential and luxury goods, such promotions can drive demand, sales and brand loyalty. However, for essential goods where need rather than supplier‐induced demand should be the main factor driving use, there is clear potential for adverse consequences. Those who would otherwise have chosen—and benefited from—breastfeeding could be encouraged to switch to formula feeding and prematurely stop breastfeeding (Michalopoulou et al. 2024). As outlined above, the Code makes clear that usual marketing practices cannot be applied to commercial milk formulas.

The proposed policy solution to allow price promotions appears particularly misplaced in a context in which the extremely high profit margins of both manufacturers and retailers exposed by the CMA remain unchallenged (Changing Markets Foundation 2017). A far simpler way to make infant formula more affordable would be to place limits on businesses' currently large profit margins (Lex 2023) so forcing a reduction in marketing spend. Even moderate controls would leave a healthy profit margin for businesses, but without the risks to public health that company‐led pricing causes.

To better safeguard the health of all UK infants, we call on the UK Government to (BFLG 2024)ictoria:

1. Actively place public health imperatives at the heart of the actions it intends to take following the CMA's final report.
2. Strengthen commercial milk formula marketing regulations and more closely align national rules with the international minimum standards on marketing of breastmilk substitutes outlined in ‘the Code’.
3. Improve the provision of sufficient, clear, authoritative and impartial advice on commercial milk formulas to counteract company advertising and better enable parents to make informed decisions on how they feed their babies and young children.
4. Impose price controls on necessary commercial milk formulas.
5. Ensure the effective enforcement of regulations.

## Author Contributions

Victoria Sibson wrote the first draft of the editorial. All authors contributed, reviewed, and approved the final version.

## Conflicts of Interest

Victoria Sibson and Catherine Pereira‐Kotze declare paid employment by First Steps Nutrition Trust, and Marko Kerac declares being a Trustee of First Steps Nutrition Trust. Robert Boyle declares payment from Wiley and the British Society for Allergy and Clinical Immunology for editorial work, from World Health Organization for consultancy, and for expert witness work in food anaphylaxis and infant formula health claim cases. Other authors declare no conflicts of interest.

## Data Availability

The authors have nothing to report.
